# Rejuvenation of brain, liver and muscle by simultaneous pharmacological modulation of two signaling determinants, that change in opposite directions with age

**DOI:** 10.18632/aging.102148

**Published:** 2019-08-15

**Authors:** Melod Mehdipour, Jessy Etienne, Chia-Chien Chen, Ranveer Gathwala, Maryam Rehman, Cameron Kato, Chao Liu, Yutong Liu, Yi Zuo, Michael J. Conboy, Irina M. Conboy

**Affiliations:** 1 Department of Bioengineering and QB3 Institute, University of California, Berkeley, Berkeley, CA 94720, USA; 2 Department of Molecular, Cell and Developmental Biology, University of California, Santa Cruz, Santa Cruz, CA 95064, USA

**Keywords:** muscle repair, TGF-beta, oxytocin, neurogenesis, neuro-inflammation, cognition, liver health

## Abstract

We hypothesize that altered intensities of a few morphogenic pathways account for most/all the phenotypes of aging. Investigating this has revealed a novel approach to rejuvenate multiple mammalian tissues by defined pharmacology. Specifically, we pursued the simultaneous youthful in vivo calibration of two determinants: TGF-beta which activates ALK5/pSmad 2,3 and goes up with age, and oxytocin (OT) which activates MAPK and diminishes with age. The dose of Alk5 inhibitor (Alk5i) was reduced by 10-fold and the duration of treatment was shortened (to minimize overt skewing of cell-signaling pathways), yet the positive outcomes were broadened, as compared with our previous studies. Alk5i plus OT quickly and robustly enhanced neurogenesis, reduced neuro-inflammation, improved cognitive performance, and rejuvenated livers and muscle in old mice. Interestingly, the combination also diminished the numbers of cells that express the CDK inhibitor and marker of senescence p16 in vivo. Summarily, simultaneously re-normalizing two pathways that change with age in opposite ways (up vs. down) synergistically reverses multiple symptoms of aging.

## Introduction

In heterochronic parabiosis, a young and old animal are surgically connected to share a common blood circulation. Experiments in mice showed this shared circulatory milieu restored tissue health and regeneration of the old partner; and at the same time, the young partner experienced a regenerative decline in a number of tissues [1–5]. In parabiosis, both organs and blood are shared, but further work focusing on exchanging only blood or infusing only plasma further detailed age-related effects on different tissues [6,7]. However, parabiosis is not clinically translatable and infusion of young blood or plasma into old mammals is controversial and fraught with multiple side-effects [1,3,6–10]. Blood fractionation is typically cumbersome, and it is inherently complicated by the fact that the rejuvenative activities are likely to be contained in multiple molecularly different fractions [11]. Plus, the assays for determining such activity are themselves complex, thus adding to the hurdles of a screen for active blood molecules. With these observations to consider, what would be the key set of molecular parameters that were changed by the blood heterochronicity and what would be best translational way forward?

The changes that manifest with aging include altered cell metabolism, increased Reactive Oxygen Species (ROS), inflammation, senescence, and decline in immune function. However, from the viewpoint of tissue maintenance and regeneration, we postulated that these arise from changes in tissue growth and homeostasis and specifically in key signaling networks regulating stem cells and their differentiated niches. In support of this idea, pathway modifier-based approaches for the enhancement of aged tissue repair and maintenance have been reported, for example, by systemic delivery of OT which induces MAPK/pERK signaling [12], by forced activation of Notch-1 [13], by antagonism of TGF-beta/pSmad signaling [14], or by antagonism of the Jak/Stat pathway [15].

The highest risk from modulating key cell-fate regulatory signaling pathways come from changing levels too far above or too far below normal healthy levels. Such drastic alterations result in severe multi-tissue side-effects. But high levels of a single modifier might be required to overcome the many age-specific molecular changes. For example, ectopic oxytocin (OT) might be needed at a considerably high dose to overcome age-elevated TGF-beta 1. And, the Alk5 inhibitor of the TGF-beta receptor might be needed at high dose to overcome the lack of OT and other hormones with age.

Using a two-prong approach of simultaneously diminishing TGF-beta signaling and adding OT (which activates pERK via the oxytocin receptor (OTR) [12]), we were able to reduce the required dose of Alk5i, shorten the duration of treatment and to achieve a more broad rejuvenation of the three germ-layer derivative tissues: brain, liver and muscle. And, we found that Alk5i+OT down-regulated the number of cells that show an age-associated increase of the cyclin dependent kinase (CDK) inhibitor and marker of senescence, p16, thereby representing a pharmacological combination of two FDA approved drugs to normalize this checkpoint protein, which when chronically elevated negatively impacts tissue health [16–22].

The translational ramifications of this study are in the attenuation and reversal of multi-tissue attrition and decline of cognitive performance in old mammals, leading to novel defined pharmacology for a number of degenerative and metabolic age-associated diseases, as a class.

## RESULTS

### In vivo delivery of Alk5i and OT

**Figure 1:**
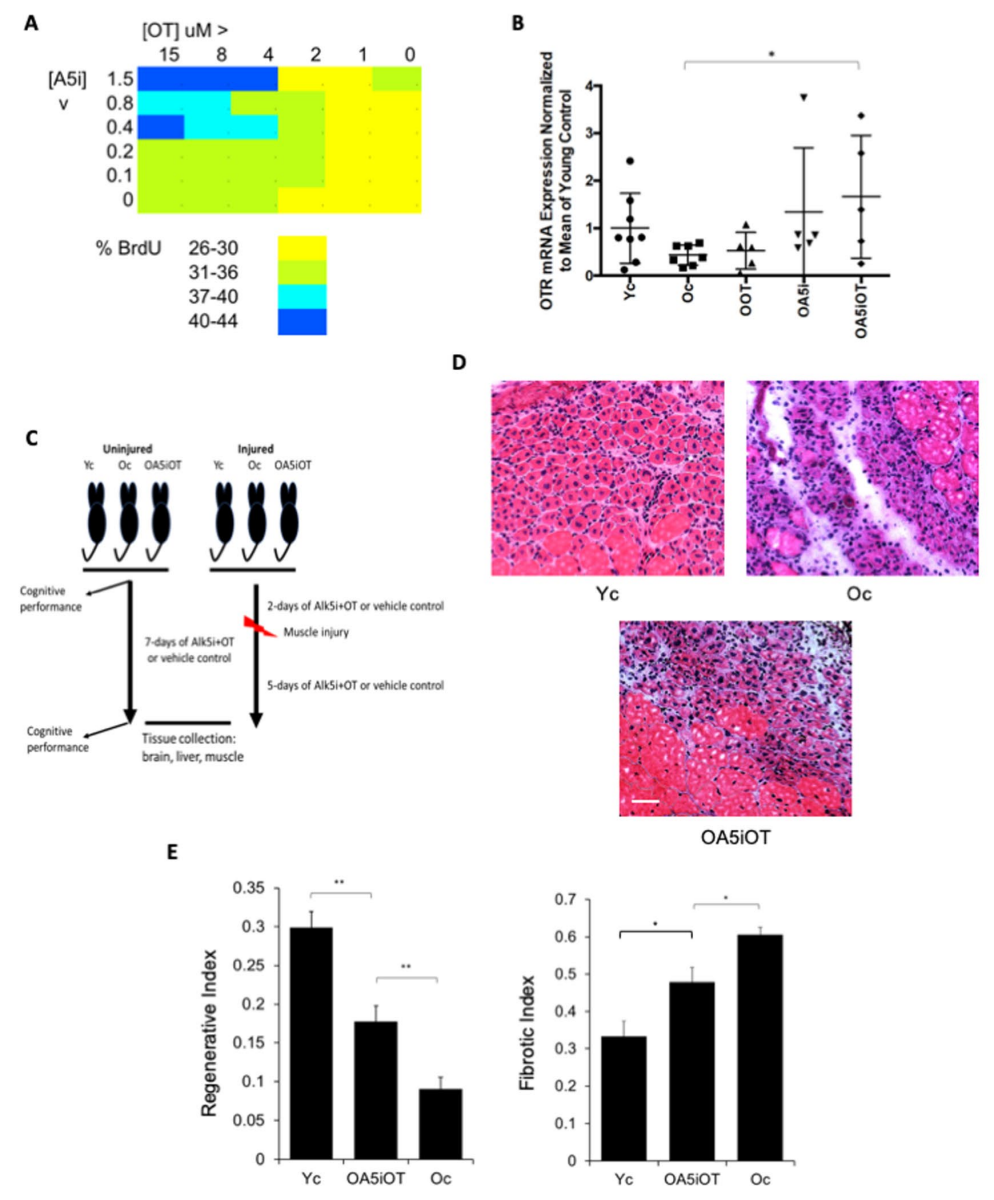
**Effects of OT and Alk5i on myogenic proliferation, OTR expression and muscle repair in vivo.** (**A**) Old muscle stem cells were freshly isolated from aged (23-24mo old) C57.B6 male mice and cultured in Opti-MEM with 5% old mouse serum. The indicated doses of Alk5i and OT (both micromolar) were added to 10^3^ cells per well of 96 well plates for 24 hours. Cells were pulsed with BrdU, immunostained and counted. Shown is the percent proliferation visualized as a heat map. The effects of OT and Alk5i on proliferation are dose dependent and at some doses Alk5i+OT has a more robust effect than each molecule alone. (**B**) Old (23-24mo) C57.B6 mice were administered by subcutaneous injections with oxytocin (OOT), Alk5i (OA5i), a mixture of OT and Alk5i (OA5iOT) or HBSS (Oc) in vivo for 7 days, daily. Young (2-3mo) mice (Yc) were injected with HBSS in an identical manner. The expression levels of oxytocin receptor (OTR) were assayed by real-time qRT-PCR in the TA muscles of these mice and were normalized to Actin. OA5iOT as compared to Oc (*p = 0.030). N = 8 for Yc, N = 7 for Oc, N = 5 for OOT, N = 5 for OA5i, and N = 5 for OA5iOT. (**C**) Schematic of the experimental procedure. Old (23-24 month) C57.B6 mice were injected subcutaneously with Alk5i+OT (0.02 nmol/g/day for Alk5i, and 1 µg/g/day for OT) (OA5iOT) or control vehicle (HBSS) (Oc) for 7 days daily. The young C57.B6 mice (Yc, 3-4 month), were identically administered with HBSS for 7 days. After two days of Alk5i+OT or HBSS injections, some young and old mice underwent experimental muscle injury and were then treated with Alk5i+OT or HBSS for 5 days; while other mice were analyzed in the absence of tissue injury. Male mice were used in these studies. (**D**) TA muscles were injured by injections of CTX and 5 days later, muscles were snap-frozen in OCT and cryosectioned to 10 µm. H&E staining was performed where newly formed muscle fibers are smaller and with central nuclei. These nascent myofibers form efficiently in the young, but not old injured muscles. As shown in representative H&E panels, Alk5i+OT dramatically enhanced in vivo myogenesis (dense areas of new myofibers) and diminished fibrosis (white areas devoid of muscle fibers). Scale bar=50 µm. (**E**) The regenerative index and fibrotic indices were defined at 5 days post CTX injury, as in (Rebo J., *et al* 2016); Alk5i+OT improved muscle regeneration and reduced fibrosis (*p=0.02201, **p Oc & OA5iOT = 0.0029, **p Yc & OA5iOT = 0.00870). N=5 for each cohort in both regenerative and fibrotic studies.

To look for synergy between Alk5i and OT, we first tested a dose curve of these compounds individually and in combination, in vitro on muscle stem/progenitor cells. Cells were freshly derived from regenerating C57.B6 mouse muscle, and cultured in old mouse serum overnight, culture conditions in which the proliferative capacity is typically low [12,23]. Alk5i and OT combined at some lower concentrations were sufficient for enhancing proliferation of these old muscle cells cultured with old serum, versus using each molecule alone (Figure 1A). This result is consistent with the OT/OTR and Alk5/TGF-beta/pSmad pathways interacting through pERK [2,11,24]. To confirm this possible mechanism, we studied the effects of Alk5i, OT and Alk5i+OT on the levels of oxytocin receptor (OTR). Interestingly, the combination of Alk5i+OT increased the expression of OTR above either drug alone or control (Figure 1B). And of note, OT and OTR signaling is needed for healthy muscle, bone, brain and metabolism [2,12].

Considering these data, we designed the in vivo study depicted in Figure 1C. We selected a combination of 1/10 the previously published [14] dose of Alk5i (0.02 nmol/g-day), and OT dosed at 1 μg/g-day by subcutaneous administration into mice to provide systemic in vivo delivery of these molecules. C57.B6 male mice at 22-24 months of age (old) were injected subcutaneously and daily with Alk5i+OT or HBSS vehicle control for seven consecutive days. As a positive control for tissue health and regeneration, C57.B6 male mice at 2-3 months of age (young) were injected subcutaneously with vehicle control, HBSS. After two days of Alk5i+OT or HBSS injections, some mice from each cohort underwent experimental cardiotoxin-induced injury to their muscle (Tibialis Anterior and Gastrocnemius) and were again injected daily with Alk5i+OT or HBSS for the 5 days of recovery (e.g., 7 days total treatment); while other mice were analyzed in the absence of tissue injury. A number of assays were performed on tissues that represent each of the three developmental germ layers, and animal cognitive capacity was evaluated, as described below.

We first tested the in vivo effects of Alk5i+OT on the regeneration of skeletal muscle, which we previously showed to be rejuvenated by either drug alone, albeit, at a 10-fold higher concentration of Alk5i [14]^,^ or upon a longer dosing with OT [12]. Tibialis anterior (TA) and gastrocnemius (Gastroc) muscle of the young and old mice were injured by cardiotoxin (CTX), and at 5 days post injury the success in muscle regeneration was determined, based on the numbers of newly-formed muscle fibers and the degree of fibrosis, as in [6,14]. The Alk5i+OT combination improved the regeneration and reduced fibrosis of the old injured muscle; and notably, this phenotype manifested at a much lower dose of Alk5i than previously published [14] and after just seven daily injections of the Alk5i+OT mix (Figure 1D, E).

### Hippocampal neurogenesis is improved, and neuro-inflammation is diminished in old mice that are treated with Alk5i+OT

Neurogenesis in the hippocampus decreases dramatically with age [25]. In previous work, heterochronic parabiosis or dosing with high level of Alk5i were shown to enhance hippocampal neurogenesis of old mice, while heterochronic blood exchange had no positive effects on old neurogenesis and profoundly attenuated young neurogenesis [2,6,7,14]. The effects of ectopic OT have not been explicitly studied with respect to age-specific neurogenesis.

**Figure 2:**
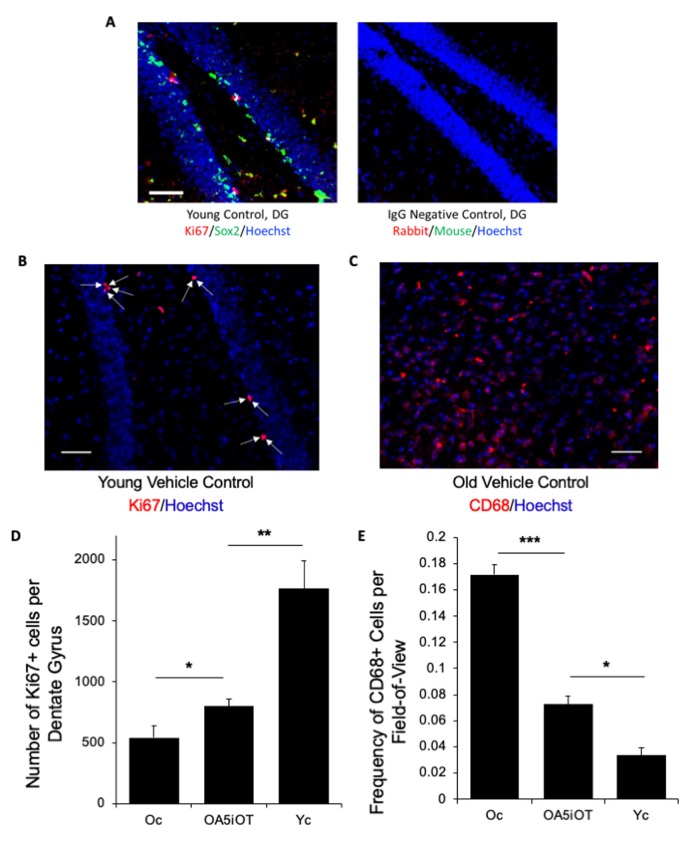
**Alk5i+OT treatment improves hippocampal neurogenesis and attenuates inflammation in the brains of old mice.** (**A**) Immunofluorescence was performed on serial 25-micron brain sections with anti-Ki67 (proliferation marker) and anti-Sox-2 (neural stem cell marker), using Hoechst to stain all nuclei. A representative image of ki67 (red)/Sox2 (green)/Hoechst (blue) triple positive cells in the hippocampal Dentate Gyrus of a young control animal treated with HBSS for 7 consecutive days is shown. Scale bar=50 µm. Isotype-matched IgG negative controls exhibited minimal background fluorescence. (**B**) Immunofluorescence was performed on serial 25-micron brain sections with anti-Ki67 (proliferation marker), using Hoechst to stain all nuclei, imaging the cells in the SGZ of the hippocampal Dentate Gyrus. Representative images of Ki67 (red)//Hoechst (blue) positive cells in the hippocampal Dentate Gyrus. Arrows point to these double-positive cells. Scale bar=50 µm. (**C**) Immunofluorescence was performed on serial 25-micron brain sections with anti-CD68 (monocyte/microglia marker), using Hoechst to stain all nuclei. Representative images of CD68 (red)/Hoechst (blue) double positive cells. Scale bar=50 µm. (**D**) The numbers of Ki67+/Hoechst+ cells in the SGZ of DG were quantified through entire hippocampi of each cohort and were found to decline with age as expected, and to increase in the Alk5i+OT old cohort, as compared to the control vehicle-treated old cohort. Young control (Yc n=5), old control (Oc n=5), Alk5i+OT (OA5iOT n=6) *p Oc & OA5iOT = 0.043, **p Yc & OA5iOT = 0.00159, mean and SE are shown. (**E**) The number of CD68+ brain cells were quantified in all cohorts and were found to increase with age and to decline in Alk5i+OT-treated old muscle, as compared to the vehicle-treated old control. N young control (Yc n=5), old control (Oc n=5), Alk5i+OT (OA5iOT n=6). ***p<0.001, **p=0.0226.

To determine whether Alk5i+OT is capable of enhancing neurogenesis in the old mice after a short 7-day treatment, we analyzed 25 micron serial brain sections for the numbers of proliferating (Ki67+) neural stem cells in the subgranular zone (SGZ) of the Dentate Gyrus (DG) of hippocampus, as published [2,6,14,26]. HBSS-injected young and old mice were used as positive and negative controls, respectively. The neural stem cell identity of the SGZ Ki67+ cells was confirmed by Sox-2 co-staining; both Sox-2 and Ki67 displayed the expected nuclear immunofluorescence, while the background of the isotype-matched IgG controls was minimal (Figure 2A). As shown in Figure 2B and quantified in 2D, Alk5i+OT when provided systemically *in vivo*, resulted in a ~ 2-fold increase in hippocampal neurogenesis of old animals in just one week. Young control mice had approximately 10-fold better SGZ neurogenesis than old controls, which is consistent with the body of published work [2,6,14,27].

One negative causal factor in the old brain that contributes to neurogenic and neuroprotective decline is an age-related increase in microglia and central inflammation locally in brain, as well as an influx of peripheral leukocytes to the brain [28,29]. To assess these phenotypes in our study, we quantified the numbers of CD68+ monocytic cells that were robustly detected in the thalamus region (Figure 2) of the brains of each studied cohort. As shown in Figure 2C, quantified in Figure 2E, roughly 8-fold more CD68+ cells were present in the old control brains as compared to young. Importantly, a 7-day treatment with Alk5i+OT reduced the numbers of CD68+ cells by ~50% in the brains of old mice, with high statistical significance.

These results establish that the low dose of Alk5i combined with OT quickly and robustly enhances SGZ neurogenesis) and reduces brain inflammation.

### Cognitive ability is improved in the old mice treated with Alk5i+OT

Cognitive decline accompanies aging in mice and humans, causing diminished ability to learn, memorize and eventually, leading to the loss of quality of life and independence. With age, neurogenesis decreases in the hippocampus – the region responsible for learning and memory, and neuro-inflammation increases, and each has been suggested as factors that contribute to the age-imposed cognitive decline [30–32].

**Figure 3:**
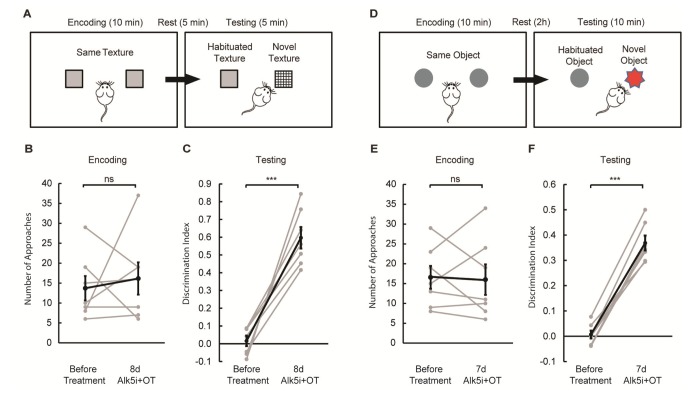
**Alk5i+OT treatment improves cognitive performance in aged mice.** (**A**) Schematics of the whisker-dependent texture discrimination task. (**B, C**) Alk5i+OT treatment does not affect the explorative behavior of aged mice during the encoding phase (**B**, *p*=0.659), but significantly improves the performance in texture discrimination (**C**, *p*<0.001). (**D**) Schematics of the NOR test. (**E, F**) Alk5i+OT treatment does not affect the explorative behavior of aged mice during the encoding phase (**E**, *p*=0.864), but significantly enhances their preference to the novel object during the testing phase (**C**, *p*<0.001). *n* = 7 for both tasks. Light grey: individual animal. Black: group average. Error bars: S.E.M. p<0.001.

Since hippocampal neurogenesis improved and neuro-inflammation diminished after Alk5i+OT treatment, we went on to explore whether treatment with these compounds would enhance the cognitive abilities of aged mice. First, we tested sensory processing using a whisker-dependent texture discrimination task (Figure 3A [33,34]. In contrast to young mice (< 2 months old), aged mice failed to distinguish between novel and familiar textures (Figure 3B, C). Such failure may be due to inability to distinguish textural differences *per se*, or due to defective short-term memory, *i.e.*, failure to remember whether a texture has been previously encountered. To assess short-term memory, we subjected aged mice to the novel object recognition (NOR) test [35], with the resting period matching that of the texture discrimination task (5 min, Supplementary Figure 1A). We found that aged mice spent significantly more time interacting with the novel object, just as young mice did (Supplementary Figure 1B). Thus, impaired tactile sensitivity or processing, rather than defective short-term memory, is likely the culprit. Next, we examined whether aged mice could retain memory by extending the waiting period in the NOR test to 2h (Figure 3D). Aged mice only approached the novel object at chance level, suggesting defective memory retention (Figure 3E, F). Henceforth we set the resting period in the NOR test to 2h.

We then treated the same group of aged mice with Alk5i+OT for 7 to 8 days and re-tested them on the texture discrimination and the NOR test. We found that the treatment did not alter the overall amount of interaction with textures and objects during the encoding phase of each task (Figure 3 B, E). Remarkably however during the test phase, the treatment significantly improved the performance of each old animal in both tasks: demonstrating preferential interest to novel texture and novel object (Figure 3 C, F).

Collectively, these data show that 2-year-old mice (equivalent to 75 to 80-year-old humans) become more competent in cognitive tasks after a week of daily administration of Alk5i+OT. The rapid cognitive improvement in old mice that were administered with A5i+OT is more likely to be associated with the diminished neuro-inflammation rather than increased neurogenesis, as it is expected take a much longer than a week for newly formed neurons to differentiate and integrate into the brain circuitry.

### Alk5i+OT reduces liver adiposity, and liver fibrosis

To examine whether Alk5i+OT is effective in liver in addition to the muscle and brain, similarly to what we found after heterochronic parabiosis and blood exchange, the above described mice that were administered for 7 days with Alk5i+OT or HBSS, were also studied for liver fibrosis and liver adiposity. As others and we previously published, in mice with age liver fibrosis and adiposity become markedly increased, and these liver health parameters are improved in old mice by decreasing or diluting the old systemic milieu and/or replacing it with young [6,23,36].

**Figure 4:**
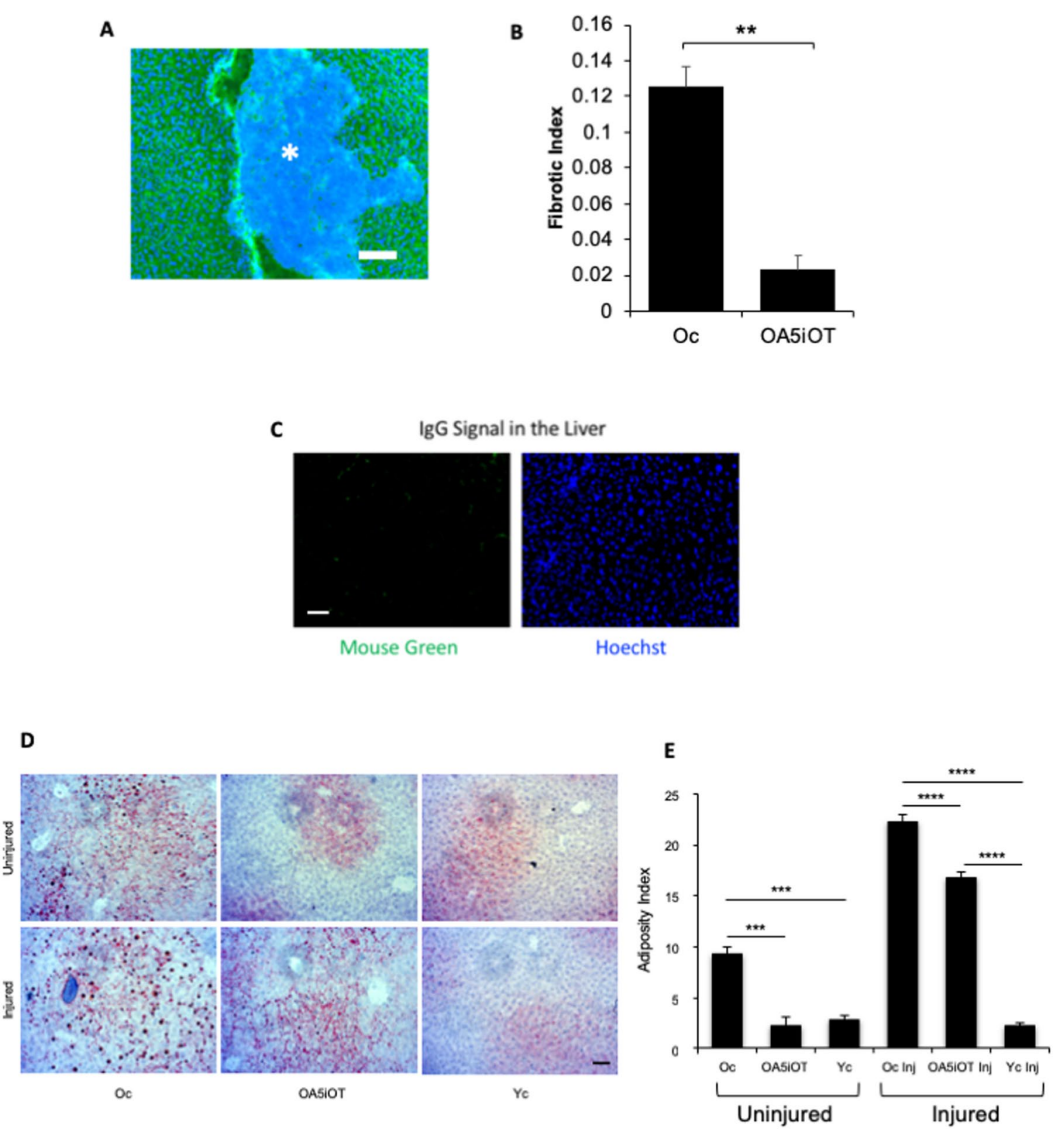
**Liver adiposity and fibrosis are reduced in old mice treated with Alk5i+OT.** Livers were collected from non-injured mice and at 5 days post cardiotoxin-induced muscle injury, as illustrated in Figure 1C. (**A**) 10 µm liver sections were immunostained with Albumin+ (green) using Hoechst dye (blue) to label all nuclei, representative image is shown. (**B**) Albumin-negative fibrotic clusters (commonly found in old, but not young livers) were quantified; the incidence of fibrosis is reduced in the livers of old injured mice that were administered with Alk5i+OT, as compared to HBSS control **p=0.001, N for old control (Oc)=3, N for old+Alk5i+OT (OA5iOT)=3. (**C**) Isotype-matched IgG signal for albumin immunodetection was minimal. (**D**) Representative Oil Red O staining of liver sections show an age-specific increase in adiposity and reduction of old liver adiposity by the Alk5i+OT in both injured and uninjured animals. (**E**) Image J quantification of red pixel density in the Oil Red O assay was performed, as published (Rebo *et al*, 2016). Alk5i+OT diminished the liver adiposity of the old injured mice. N=6 in each injured cohort, ****p= 0.002 Oc & OA5iOT, N=6 in each uninjured cohort **p<0.001 Oc & OA5iOT. All scale bars=50 µm.

To evaluate the liver health, we performed immunofluorescence for albumin on 10-micron liver sections and quantified the relative number of albumin-negative fibrotic clusters. Additionally, liver adiposity was measured by Oil Red O staining in these tissue sections, as in [6,23]. As shown in Figure 4, the low dose of Alk5i combined with OT within one-week reduced the adiposity and fibrosis of old livers, making these more similar to control young mice. Additionally, muscle injury increased liver adiposity in the old mice (Figure 4).

These results demonstrate that defined pharmacology of Alk5i+OT exhibits positive effects not just on brain or skeletal muscle, but also on liver health in old mice; and to a similar extent as reported for heterochronic parabiosis and blood exchange.

### Alk5i+OT attenuates the numbers of p16^+^ cells in multiple tissues of old mice in vivo

Considering the enhanced myogenesis and neurogenesis that we observed in the old mice, which were administered with Alk5i+OT, we decided to have a closer look at the effects of this two-molecule pharmacology on the CDK inhibitor and marker of senescence, p16. An increase in p16-expressing cells is thought to be deleterious, particularly in old age [19,37,38], and knocking out p16 enhances adult stem cell performance in multiple tissues [18,20,21]. The levels of p16 and other CDK inhibitors, p15, p21 and p27, increase with age in muscle stem cells, interfering with productive regeneration [39–41]. OT has been shown to down-modulate the CDKI p21 in a pERK-dependent manner [12], but the effects on p16 were not studied. Similarly, at a high dose, Alk5i attenuates p21 levels in brain of old mice [14], but p16 was not studied.

**Figure 5:**
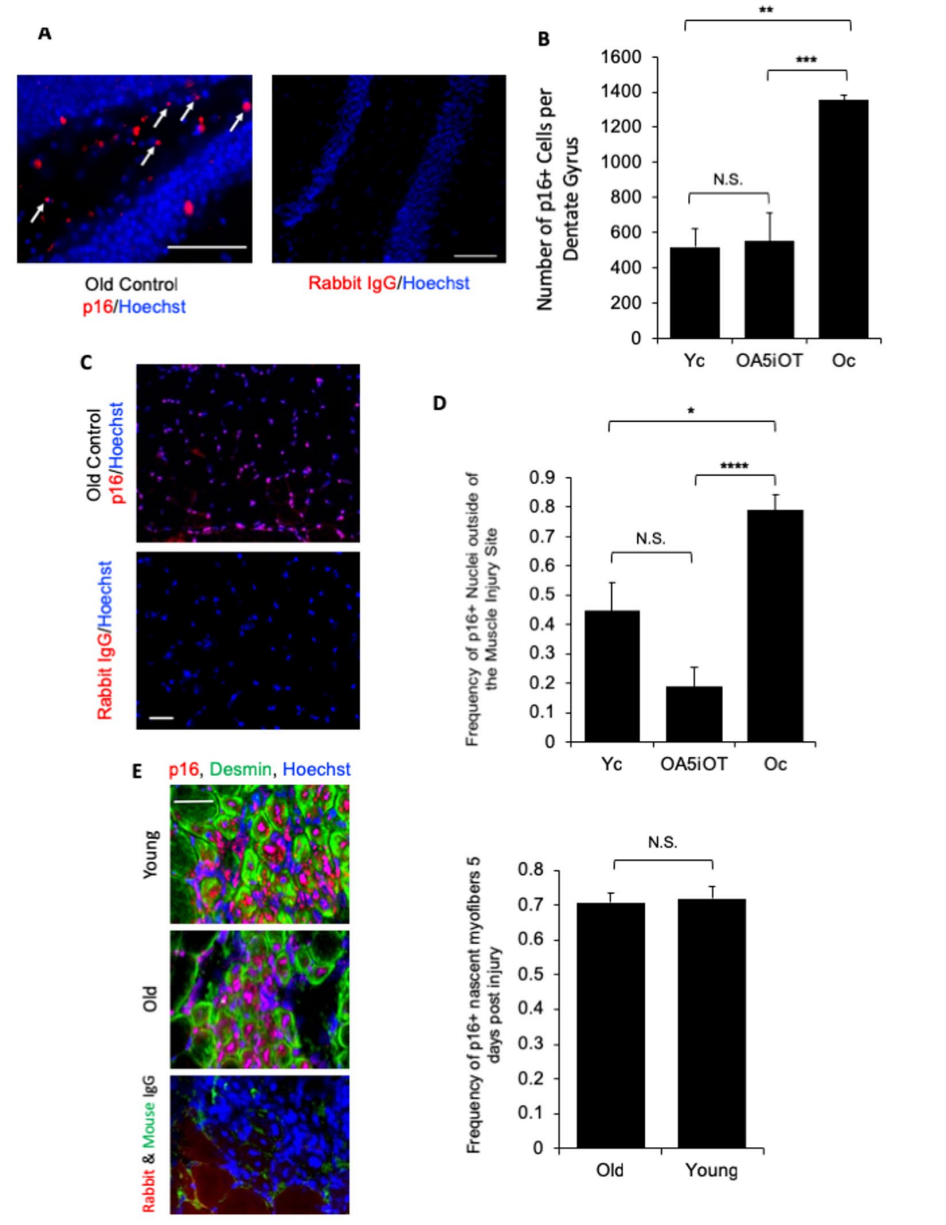
**p16 levels are decreased *in situ* in muscle and brain by Alk5i+OT.** Cryosections of injured and uninjured muscle (10 µm, each) and brain (25 µm) were assayed for the number of p16+ nuclei by immunofluorescence, using Hoechst to label all nuclei. p16 showed the predicted nuclear localization in these assays. (**A**) Representative images of p16+ cells in the polymorphic layer near the dentate gyrus in an old control brain and isotype-matched IgG non-specific immunofluorescence are shown. Arrows point to p16+ (red)/Hoechst+ (blue) nuclei in the stated region. (**B**) The number of p16+ cells in the polymorphic layer of the hippocampus was decreased by the Alk5i+OT treatment, ***old control & Alk5i+OT p=0.0003, scale bar=50 µm at 40x magnification, IgG scale bar=50 µm at 20x magnification. Young control (Yc) n=5, old control (Oc) n=8, Alk5i+OT (OA5iOT) n=8. (**C**) Representative images of p16+ nuclei outside of the injury site in the TA muscle of an old vehicle control mouse at 5 days post single CTX injection and isotype-matched IgG non- specific immunofluorescence, are shown; scale bar=50 µm at 20x magnification. (**D**) The number of p16+/Hoechst+ nuclei divided by the total number of nuclei (Hoechst+) per field-of-view at 20x magnification (frequency of p16+ nuclei) was quantified. The frequency of p16+ nuclei outside of injury sites is significantly greater in muscle of old vehicle-treated control mice, as compared to young control and old Alk5i+OT treated. N=5 for each cohort. Young control (Yc), old+Alk5i+OT (OA5iOT), old control (Oc) N.S. = P-value Yc &OA5iOT = 0.064, *** = P-value Yc & Oc = 0.011, **** = P-value OA5iOT & Oc = 0.000064. (**E**) Representative images of the sites of injury/regeneration of young and old TA muscle from control - HBSS treated mice; 10-micron sections desmin (green), p16 (red) immunofluorescence and isotype-matched IgG control non-specific immune-fluorescence, are shown. Scale bar is 50 micron at 40x magnification. Robust p16+ nuclei are observed in both young and old muscle, and many of these are in centrally-nucleated newly formed desminhigh myofibers. The frequency of p16+ centrally-nucleated myofibers was quantified (right). The relative number of these p16+ fibers were found to be nearly identical in young injured (n=5) and old injured (n=7) muscle. N.S. = P-value Old and Young = 0.7918.

We examined the effects of in vivo administered Alk5i+OT on the number of p16^+^ cells in brain, muscle, and liver by p16-specific immunofluorescence on tissue sections. The p16 signal was nuclear as expected, and immunofluorescence from isotype-matched IgG controls was negligible (Figure 5). As shown in representative images, p16^+^ mono-nucleated cells were observed in the polymorphic region of the hippocampus near the Dentate Gyrus (Figure 5A), and the numbers of these p16 expressing cells were elevated in old mice as compared to young, and reduced in old animals that were administered with Alk5i+OT, as compared to the vehicle-treated old mice (Figures 5B). As compared to young muscle, higher number of p16+ mono-nucleated cells were also found in old TA muscle, outside of the injury sites; and the numbers of these p16+ cells declined in old mice that were administered with Alk5i+OT (Figure 5C and D).

Interestingly, during these studies we have detected significant numbers of p16^+^ cells in both young and old muscle at the injury/regeneration sites, at 5 days post injury, a time point when proliferating myoblasts typically differentiate into new myofibers (Figure 5 E). Indeed, the p16 immunofluorescence was found in a majority of newly-formed centrally-nucleated desminhigh myofibers in young and old tissue and there was no statistically-significant age-specific difference in the numbers of newly-formed p16+ muscle fibers (Figure 5E). Co-immunodetection of p16 and laminin was also performed to confirm the findings with p16 and desmin (Supplementary Figure 2). We did not detect p16^+^ cells in the livers of either young or old mice by the same immunofluorescence assay.

These data show that cells which express p16 become elevated with age, as expected from the body of published work [19,37,38,42], and suggest that the seven-day Alk5i+OT treatment reduces the numbers of p16^+^ cells in brain and in muscle of old mice. These data also demonstrate that p16^+^ myofibers are a normal part of physiological regeneration of injured muscle in both young and old.

## DISCUSSION

This work demonstrates a reversal in multiple tissues, of several signs of aging, through the simultaneous modulation of two signaling pathways, one of which becomes elevated with age and the other reduced. Translationally, this study points toward a pharmacological approach to rapidly enhance the health and maintenance of multiple old tissues. Here we focused on a few key age-related parameters of the three germ layer tissues: neurogenesis and neuroinflammation of the brain, regeneration and fibrosis of the skeletal muscle and adiposity and fibrosis of the liver. In future work if would be interesting to study how these seemingly unrelated aging features become rapidly rejuvenated by A5i+OT, and if additional phenotypes, such as muscle innervation, neural plasticity, metabolism, etc. also become improved in old animals. The observed rejuvenating effects are at least as robust as, and act faster than, heterochronic parabiosis [2,23]. Additionally, the effects of Alk5i+OT are more positive for neurogenesis and restore the functional behavior of old mice, as compared with heterochronic blood exchange [6].

OT and various Alk5 inhibitors [43] are already FDA approved for applications that are different from age-related degenerative pathologies, and hence repositioning this combination is facilitated. OT is not associated with cancers or any other pathologies, and in fact a lack of OT is known to cause depression, obesity, osteoporosis and muscle wasting [12,44–46]. Alk5 inhibitors are in clinical trials for combating cancer progression, because at high levels TGF-beta switches from inhibiting to promoting cancer metastasis, and from attenuating to promoting inflammation [14,47,48]. Of note, both Alk5i and OT have been shown to cross the blood brain barrier, enabling delivery to all organs and tissues [14,49].

The effects of Alk5i+OT on neurogenesis and myogenesis implicate simultaneous and rapid positive changes to the tissue specific stem / progenitor cells and their niches. The reduction of fibrosis in muscle and liver represents another global feature of organ aging that is reversed by the Alk5i+OT. The observed reduction in liver adiposity might be indicative of general metabolic improvements in the old mammals. There is a good consensus on the dramatic age-related decline in muscle stem cell proliferation after tissue injury or attrition. Approaches that enhance satellite cell proliferation improve not just myogenesis, but also tissue health and strength of old animals without any cell transplantation [1,12]. Aged stem cells have more than adequate regenerative potential, and enhancement of their proliferation leads to positive outcomes [1,12,14]. This notion is expanded in current work.

Central neuro-inflammation, with its characteristic swollen microglia expressing CD68, is not only causal to the age-related loss of neurons [50], but also implicated in psychiatric diseases such as schizophrenia [51]. In this regard, the observed attenuation of CD68+ brain cells by Alk5i+OT might also be clinically relevant on several fronts.

It is likely that the rapid cognitive improvement in aged mice results from reduced neuro - inflammation, rather than from the increased neurogenesis. First of all, there was no time in our study for newly-formed neuronal cells to mature and integrate into the brain networks, as we assayed cognitive performance after just 7 days of Alk5i+OT administration. Secondly, prior studies have suggested that neuro-inflammation induced by heat stress severely impairs the performance on New Object Recognition tests (the cognitive skill that were examined in our work), and the administration of minocycline, an anti-inflammatory agent, inhibits heat stress-induced cognitive deficits and astrogliosis [52]. Attenuated neuro-inflammation might reflect a direct effect of our pharmacology on these myeloid lineage cells, an indirect effect through diminished peripheral and/or central inflammation, or both. At high dosage, Alk5i alone reduces the levels of the inflammatory marker B2M [14], but the low dose of Alk5i that is combined with OT increases the levels of OTR in old animals, and OTR signaling is needed for the health of the brain, as well as, peripheral tissues. Thus, a low dose of Alk5i that is combined with OT has greater potential clinical advantages.

Other considerations for not overly diminishing TGF-beta signaling include the need for this pathway in the balance between tissue damage and host defense [53], immune modulation through attenuation of MHC genes and concomitant activation of NK cells [54,55], and regulation of proliferation, differentiation, and growth of various cell types [20]. These reasons and our experimental observations in sum support the notion of minimizing the dose of pathway modulating drugs such as Alk5i, which as shown here, does not restrict but broadens the positive multi-tissue effects through combination with OT. OT alone has been shown to enhance the regeneration of old injured muscle in a 9-day protocol [12]. However, we shortened the treatment to 7 days and expanded the positive outcomes to brain and liver, by combining OT with Alk5i.

One mechanism which might be involved in the rejuvenative effects of Alk5i+OT, is the reduction of numbers of p16^+^ cells in old tissues. High p16 generally corresponds with senescent cell phenotypes, and there is a profound interest in development of new pharmaceuticals for p16^high^ cell ablation (senolytics) or p16 down-modulation (senomorphics) as a way to increase life-span and/or health-span [16,38,42,56–59].

Alk5i+OT is needed at a much lower dose and for much shorter time than another molecule, ABT263, a Bcl-2 inhibitor that has senolytic properties (and relatively higher toxicity) [16]. Recently, a synthetic peptide named FOXO4-DRI was shown to interfere with canonical FOXO4-p53 interaction, consequently inducing apoptosis in senescent cells [60]. Though promising, this peptide is not FDA approved and its toxicity effects, acute or chronic, are not known. Further in contrast to our present findings, its positive effects on neurogenesis, muscle, and liver were not reported.

Importantly, our work suggests that p16 elevation at the time and place of de-novo myofiber formation might represent a required physiologic response (similar to a previous report on skin wound healing [61]. Indeed, when normalized by the total de-novo centrally-nucleated myofibers, the numbers of those with p16^high^ myonuclei that regenerated tissue injury were nearly identical between the young and old mice. Thus, ablation of all p16-positive cells could be a risky therapy; in contrast, a moderate, youthful re-normalization should be safer.

In summary, the new paradigm of this work is that individual detriments of aging might have a common cause: the concordant alteration of a few signal transduction networks, and points to a rational strategy of re-calibrating a few key pathways for combatting many age-related diseases simultaneously, as a class.

## MATERIALS AND METHODS

### Animal experiments

### *Animals*

All procedures were performed in accordance with the administrative panel of the Office of Laboratory Animal Care, and the protocol was approved Animal Care and Use Committee. Young male C57BL/6 mice were purchased from the Jackson Laboratory (#00664). Old male C57BL/6 mice (20-24 month) were purchased from the National Institute on Aging.

Mice were housed at UC Berkeley and maintained on 2018 Teklad Global 18% Protein Rodent Diet: Crude Protein 18.6%; Fat (ether extract); 6.2%; Crude Fiber 3.5%; Energy Density 3.1 kcal/g 13.0 kJ/g. Calories from Protein 24%, Calories from Fat 18% Calories from Carbohydrate 58%.

### Oxytocin (OT)

Was purchased from Bachem (H-2510) and a 30mM stock prepared in sterile water.

### *Alk5 inhibitor (A5i)*

TGF-β1 Type I Receptor Kinase Alk5 inhibitor 2-(3-(6-Methylpyridin-2-yl)- 407 1H-pyrazol-4-yl)-1,5-naphthyridine, was purchased from Enzo Life Sciences, and a 25 mM concentrated stock dissolved in DMSO. OT and/or A5i or control vehicle (HBSS) was injected subcutaneously to old male C57.B6 mice (23-24 month), daily for 7 days before sacrifice. 3 month C57.B6 male mice identically injected with HBSS were used as young controls.

### *Cardiotoxin muscle injury*

Two days after the start of oxytocin and/or Alk5 inhibitor or control, mice were injured by intramuscular injections of cardiotoxin (Sigma, 10 μl per muscle at 0.1 μg/ml) into the tibialis anterior (TA). Five days after the injury, TA muscles were isolated.

### *Cultures of muscle satellite cells*

Myofiber-associated satellite cells were isolated from TA and Gastrocnemius muscles of aged (22-24mo old) C57.B6 male mice and plated at 103 cells per well of 96 well plates in Opti-MEM with 5% old mouse serum. Alk5i and OT were serially diluted to the cell wells ranging from 0-1.5 and 0-15 micromolar, respectively, and cells were cultured for an additional 24 hours. Cells were pulsed with BrdU for 4 hours, immunostained and counted.

### *Primary and secondary antibodies*

Were used at 0.5-1 μg/ml as follows:

-Albumin: R&D Systems, Mouse, MAB1455, 1:1000

-Beta-actin: Thermo Scientific MA5-15739

-BrdU: Abcam ab6326 (BU1/75)

-CD45: Rat, Clone F10-89-4, EMD Millipore 05-1410, 1:500

-Desmin: Sigma-Aldrich, Mouse, DE-U-10, 1:300

-Laminin: Santa Cruz Biotechnology, Rat, sc-59854, 1:200

-Ki67: Abcam, Rabbit, ab16667, 1:200

-p16: Abcam, Rabbit, 189034

-Sox2: Santa Cruz Biotechnologies sc-17320, 1:400

-Isotype-matched mouse, rat and rabbit IgG’s were used as negative controls (Sigma Aldrich), 1 μg/ml

-Goat anti-rabbit Alexa 546: Invitrogen A11010, 1:2,000

-Goat anti-mouse Alexa 488: Invitrogen A11029, 1:2,000

-Donkey anti-rat Alexa 488: Invitrogen A21208, 1:2,000

-DNA was stained by Hoechst DNA dye at 1 μg/ml: -Hoechst 33342 from Sigma Aldrich (B2261)

### *Behavioral tests*

The whisker-dependent texture discrimination test was performed as previously described [34], with the encoding, resting, and testing phase lasting 10, 5, and 5 min, respectively. The novel object recognition (NOR) test was performed as previously described [34,35] with the encoding and the testing phases both set to 10 min. Such modifications of encoding and testing duration are intended to accommodate the slow movement of aged mice. Two resting periods (5 min and 2 h) were used for the NOR test in two separate sessions on the same mouse. Behavioral analyses were performed with the analyst blinded to the identity and the conditions (age, resting period, pre/post-treatment) of the mice.

### *Tissue isolation*

Was performed postmortem. Brains from uninjured animals were harvested and incubated in 4% paraformaldehyde (PFA) overnight at 4^0^C and subsequently stored in 30% sucrose. Brain, liver, and muscle harvested from injured animals and muscle and liver from uninjured animals were snap-frozen in isopentane (-70^0^C) and embedded in Tissue-Tek Optimal Cutting Temperature (OCT, Sakura Finetek, The Netherlands).

### *Tissue sectioning, and immunofluorescence*

Tissue sectioning, and immunofluorescence of brain, muscle, and liver sections were directly collected on positively charged frosted glass slides. Brain, liver, and muscle sections were 25 μm, 10 μm, and 10 μm respectively. Tissues were fixed with 70% ETOH overnight (muscle and liver) or 4% PFA for 10 minutes (brain) and blocked with 1% bovine growth serum (BGS, Hyclone), in 1X PBS for 30 minutes at room temperature. They were subsequently permeabilized with 0.1% Triton-X 100 for 10-15 min on ice, incubated with primary antibodies at 4^0^C overnight in PBS+1%BGS, washed in this buffer and incubated with secondary fluorochrome tagged antibodies and Hoechst for 2 hours at RT. IgG controls with isotype-matched antibodies were routinely done and non-specific fluorescence was minimal.

### *Hemtoxylin and eosin staining*

Was performed on tibialis anterior muscle sections that were 10 μm thick. This assay was performed as previously described [6].

### *Oil red staining*

10-micron liver sections were hydrated in 1X PBS for approximately 10 minutes. The sections were then washed in 60% isopropanol for 5 minutes and later placed in isopropanol-based Oil Red O staining solution for 15 minutes. After, the sections were washed in 60% isopropanol once more for 5 minutes. Nuclei on these sections were stained by a 1-minute wash in hematoxylin. The sections were finally washed in deionized water for one minute. Fluoromount was used as the mounting medium and images were taken from these slides.

### *qRT-PCR analysis*

Tissue lysates from frozen muscle embedded in OCT were prepared by collecting 12 slices of 50 µm thick sections. Tissues were homogenized and total RNA was extracted with RNeasy Mini Kit (QIAGEN) according to manufacturer’s instructions. Reverse transcription was performed with Superscript III First-Strand Synthesis System (Invitrogen). Real-time PCR was performed using iQ™ SYBR Green Supermix (Bio-Rad) under CFX Connect Real-Time System (Bio-Rad) and reactions were run in triplicates. 200 ng of total cDNA were used for the initial amplification using specific primers to each gene of interest. Amplification was performed with a denaturation step at 95°C for 3 minutes, followed by 50 cycles of denaturation at 95°C for 10 seconds and primer extension at 60°C for 30s. Melt curves were analyzed and samples with peaks indicating improper products were eliminated. Housekeeping gene actin was used as an internal control to normalize the variability in expression levels, and results were analyzed via Gene Study with CFX Manager Software. One outlier was eliminated because it fell further from the mean than 1.5 times the interquartile range (in the Oc cohort).

### *Primers*

-actin-f: CGCCACCAGTTCGCCATGGA

-actin-r: TACAGCCCGGGGAGCATCGT

-OXTR-f: GATGTCGCTCGACCGCTG

-OXTR-r: CGGTACAATGTAGACGGCGA

### Data quantification

At least five independently treated animals per each cohort (young control, old control, old + Alk5i, old +OT, old + Alk5i+OT) were used in quantification for each tissue; and such assays (qRT PCR, mmunofluorescence, functional performance test) were performed in 3-4 replicates. Student two-tail t-test and/or ANOVA were performed for analyzing the data quantification, and p-values of 0.05 or less were considered statistically significant.

The animal’s performance in the NOR test and the whisker-dependent texture discrimination task was quantified by calculating a discrimination index as previously described [34]. Muscle regeneration indices were calculated by counting percent de-novo myofibers with central nuclei to total nuclei in multiple images of 10-micron muscle sections for independently treated animals.

Muscle fibrosis was quantified by measuring fibrotic areas on Image J. These fibrotic areas were normalized to the area of the image taken at 20X (~ 14000 micron^2).

Hippocampal neurogenesis (Ki67+), p16+ SGZ cell numbers and CD68+ cell numbers were quantified by counting the number of respective cells (from multiple 25-35-micron IF brain sections). The number of cells positive for Ki67, p16, or CD68 were extrapolated to the total thickness of the regions where they were identified (~2.8 mm for dentate gyrus and ~2.2 mm for thalamus).

Counting cell numbers from multiple 10-micron sections for each cohort produced the quantification of albumin+, and Hoechst+ cells (hepatocytes) and of albumin- fibrotic cells per area in images obtained at 20X. Oil Red O quantification was done in 10-micron liver sections, by obtaining the total area of the red fatty droplets as a percentage of the entire 20X image area, using a consistent color-threshold function in software ImageJ.

The number of p16+ nuclei in brains and in injured and uninjured muscle was divided by the total number of nuclei present in their respective field of views; for injured muscle, it was divided by the number of myonuclei in desmin+ myofibers, at 20X magnification.

## Supplementary Material

Supplemental Figures
