# Supplementary material for: Rejuvenation of brain, liver and muscle by simultaneous pharmacological modulation of two signaling determinants, that change in opposite directions with age
Source: Aging (Albany NY). 2019 Aug 15;11(15):5628–45. doi: 10.18632/aging.102148 (PMC6710051; doi:10.18632/aging.102148)
Supplement: Supplemental Figures [file aging-11-102148-s001.pdf]

SUPPLEMENTARY FIGURES

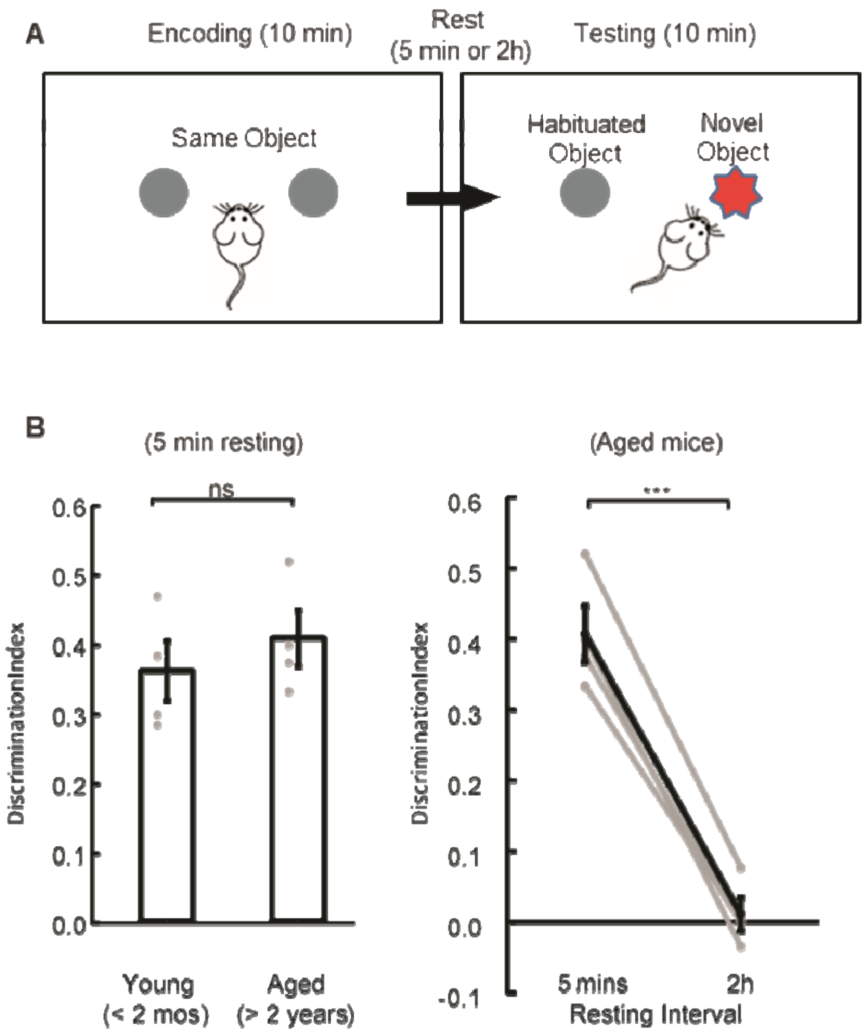

Supplementary Figure 1. Varying resting period results in different performance of NOR test in aged mice. (A) Schematics of the NOR test, two resting time periods were tested. (**B**, **C**) Aged mice perform similarly to young mice under 5 min rest (**B**, p=0.455), but fail to recognize the novel object following 2 h rest (**C**, p<0.001). Light grey: individual data points. Black: group average. Error bars: S.E.M. *** p<0.001.

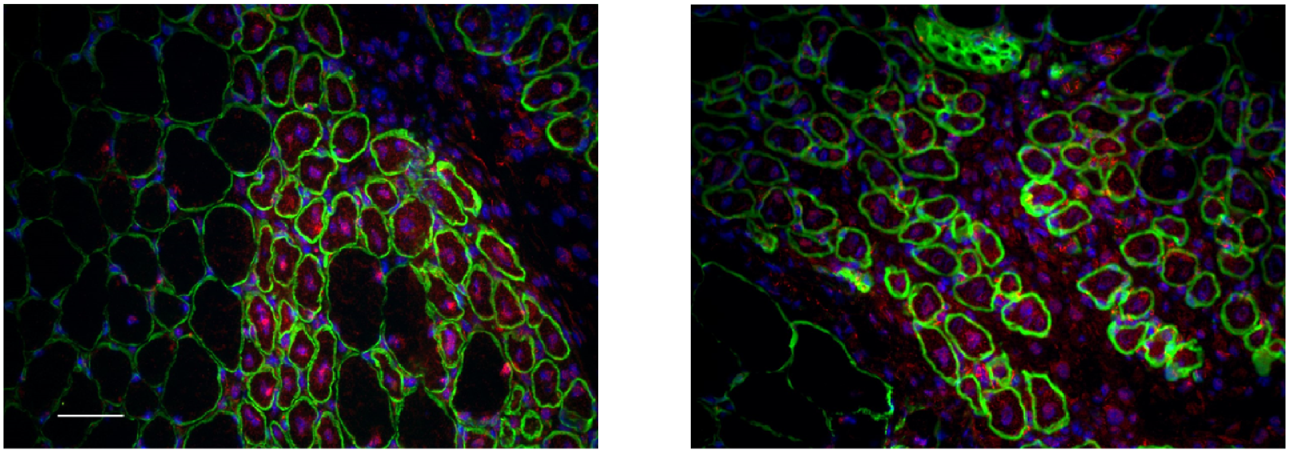

Young Old

Supplementary Figure 2. Representative images of p16/laminin immunofluorescence at young and old muscle injury sites 5 days post injury. Laminin+ (green) nascent myofibers of young and old muscle concomitantly expressing p16 (red) against Hoechst DNA dye (blue).
